# Cholera

**Published:** 1854-11

**Authors:** 


*Cholera.*—In entire disregard of a statement in our leading editorial, that the last case of cholera for the season had occurred in this city, a few cases have happened very late in October. On Friday the 27th ult., we were called to a man who had fallen in the street in collapse. As sometimes happens he had kept about the street until attacked by cramps. He was pulseless at the time we saw him. He was removed to the hospital, put under Dr. Newman's charge, his head shaved, and the ice-cap applied, and at the last accounts was convalescing. H.

